# A two‐point scheme for optimal breast IMRT treatment planning

**DOI:** 10.1120/jacmp.v14i6.4525

**Published:** 2013-11-04

**Authors:** Weiguang Yao

**Affiliations:** ^1^ H. Bliss Murphy Cancer Centre Eastern Health St. John's NF Canada

**Keywords:** breast IMRT, optimal treatment planning, optimal beam weight

## Abstract

We propose an approach to determining optimal beam weights in breast/chest wall IMRT treatment plans. The goal is to decrease breathing effect and to maximize skin dose if the skin is included in the target or, otherwise, to minimize the skin dose. Two points in the target are utilized to calculate the optimal weights. The optimal plan (i.e., the plan with optimal beam weights) consists of high energy unblocked beams, low energy unblocked beams, and IMRT beams. Six breast and five chest wall cases were retrospectively planned with this scheme in Eclipse, including one breast case where CTV was contoured by the physician. Compared with 3D CRT plans composed of unblocked and field‐in‐field beams, the optimal plans demonstrated comparable or better dose uniformity, homogeneity, and conformity to the target, especially at beam junction when supraclavicular nodes are involved. Compared with nonoptimal plans (i.e., plans with nonoptimized weights), the optimal plans had better dose distributions at shallow depths close to the skin, especially in cases where breathing effect was taken into account. This was verified with experiments using a MapCHECK device attached to a motion simulation table (to mimic motion caused by breathing).

PACS number: 87.55 de

## I. INTRODUCTION

Intensity‐modulated radiation therapy (IMRT) utilizes dynamic MLC motion to spare surrounding normal tissues and achieve better dose conformity and uniformity within the target. Many researchers have investigated the use of IMRT or hybrid (unblocked beams plus IMRT segments) techniques for the treatment of breast cancer.^1^, ^2^, ^3^, ^4^, ^5^, ^6^, ^7^, ^8^ The unblocked beams in this paper refer to those nonwedged but shaped by MLC leaves and jaws to cover the whole breast/chest wall, and the 3D CRT plans refer to plans composed of such unblocked beams and field‐in‐field segmented beams from forward planning. One of the essential issues in breast IMRT is the breathing effect. Breathing will equivalently smooth out the modulated radiation fluence map and thus change the planned dose distribution. Furthermore, breathing may be hard to control and not periodic,^(9)^ and may interplay with MLC leaf motion when the sliding window technique is used to deliver dose.^10^, ^11^ However, phantom studies where doses delivered with and without breathing were compared, have shown that dose distribution discrepancy caused by breathing is similar between 3D CRT and IMRT, based on the statistical analyses such as gamma test, and mean and standard deviation.^(12)^ The main discrepancy is located near the skin, where breathing effect is more pronounced in IMRT than in 3D CRT plans.^(12)^ This is mainly because in 3D CRT plans, usually more than 90% of the dose is delivered via unblocked beams (including at least 2 cm air gap from the skin), while in IMRT plans, because objective‐based inverse optimization algorithms are used, radiation fluence is restricted to be within the body.

As the breathing amplitude increases, the breathing effect becomes more severe.^9^, ^10^ Skin flash technique, available in some commercial treatment planning systems (TPS), can be used to mitigate breathing effect, but it is not optimized. In context, we refer skin flash as the technique to generate fluence in the air gap from the skin.

For intact breast, the IMRT treatment plans (i.e., neither breathing effect nor setup uncertainty is included) inherently have more skin sparing than 3D CRT plans. This is because the planning target volume (PTV) (an objective in the inverse planning) does not include the skin region and is a few millimeters away from the skin in order to avoid a large number of monitor units dumping into the skin region. The dumping usually has no clinic benefit because of the breathing and setup uncertainty. In addition, compared with the field‐in‐field segment beams in 3D CRT, IMRT beams cost more monitor units that may increase probability of radiation induced tumor in the contralateral breast.

Hybrid planning uses unblocked beams and IMRT beams to mitigate these concerns by increasing the weights of unblocked beams.^12^, ^13^ Optimization of the weights, however, has not been proposed in the literature. For breast and chest wall treatments, there are some special properties that can be used to optimize the plans. First, in order to limit lung dose, the beams are usually tangential in the lateral and medial directions, unlike other IMRT treatments such as prostate and head‐and‐neck treatments where multiangle beams are employed. Second, the moving target (the breast or chest wall) is located near air, so that if the IMRT fluence is close to zero in the skin region, the breathing effect can be minimized. Third, usually only dose to ipsilateral lung is the major concern, although dose to other organs, such as heart, is also of concern in left‐sided breast cancer treatments. On the other hand, unlike other site IMRT plans where only low‐energy beams are used, breast plans often need high‐energy unblocked beams to reach dose uniformity, especially for large breasts and chest walls. This is because the breast IMRT beams are limited in the lateral and medial directions. However, use of high‐energy unblocked beams results in more skin sparing and neutron dose, and thus the beam weights of high‐energy unblocked beams should be minimized. In this work, we propose a treatment planning approach to assigning optimal weights to low‐ and high‐energy unblocked beams, as well as to IMRT beams, in order to effectively decrease the breathing effect and to keep skin dose as much as possible when the skin is included in the target, or otherwise to keep skin dose as little as possible if the skin is excluded from the target.

## II. MATERIALS AND METHODS

### A. The optimization scheme

**Figure 1:**
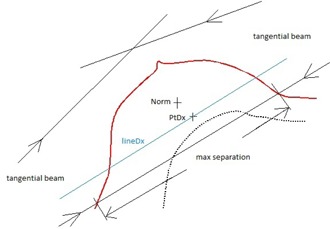
Illustration of the setup of beams, definition of maximum separations, locations of points “Norm” and “PtDx”, and line “lineDx” used in our optimal scheme. Note that the maximum separations, “Norm” and “PtDx”, are not necessary on the same axial plane in CT images.

Our hybrid plan consists of tangential high‐energy unblocked beams (i.e., 18 MV) and low‐energy unblocked and IMRT beams (i.e., 6 MV). The study is to determine the optimal weights for these beams. If the weight is zero, the corresponding beams will not be actually used. For a certain dose prescription, in order to decrease breathing effect, the unblocked beam weights should be assigned as high as possible. This may also decrease the total number of monitor units. Further, in cases where the skin is also the target, the weights for the low‐energy beams should be assigned as high as possible, provided that the dose uniformity is kept — for example, the target dose within 95% and 105% of the prescription dose. Use of bolus may relax the requirement of low‐energy beams, but in order to limit the neutron dose, the weights of high‐energy beams should be minimized. Finally, between the low‐energy unblocked and intensity‐modulated beams, again as high as possible weights should be assigned to the unblocked beams such that the photon fluence in the IMRT beams is close to or equal to zero in the skin region. Thus, the breathing effect in the skin region can be minimized. By combining these considerations, for a certain breast/chest wall treatment, we use the following steps to determine the optimal weights of the low‐energy unblocked beams, high‐energy unblocked beams, and low‐energy IMRT beams. Please refer to Fig. 1 for the setup of beams and parameters used in our optimal approach.

Step 1: Set up two unblocked beam preplans with defined tangential fields for 3D CRT, one for low energy and the other for high energy. We shall use these unblocked beam preplans to calculate the optimal weights for those beams in the final hybrid plan. The unblocked beam geometry (including the beam isocenter) is exactly the same as that in 3D CRT plans. For instance, half beam technique is used if the supraclavicular nodes are involved. Usually, the gantry angles in the low‐energy beams are the same as in the high‐energy ones; the fields are conformal to the target by using MLC and collimators, and have about 3 cm air gap from the skin; and the tangential posterior field edges are matched to spare lung dose. The target is defined based on RTOG 1005, and wires are usually used during planning CT scans to help identify the target in CT image.

Step 2: For each preplan, calculate and normalize the dose with 100% prescription at the center of target. Adjust the weights of tangential beams to balance the dose distribution along the tangential direction, namely, to obtain as much uniform dose distribution along the tangential direction as possible. The normalization point, “Norm”, is not necessary at the center of target, but should not be in the beam penumbra region (i.e., at least 1 cm away from lung and air and 2.5 cm away from the posterior field edge or shielding).

Step 3: From the dose distribution in the low‐energy unblocked beam preplan, find a point, “PtDx”, at which the dose is minimal but at least 1 cm away from lung and air, and 2.5 cm away from the posterior field edge or shielding. The minimal dose point can be found from isodose lines, or even more conveniently from dose color wash. The point is often closer to the posterior field edge and the center of the target on this 2D axial slice, as indicated in Fig. 1. (See later for the sensitivity on the optimal weight and the final dose distribution if “PtDx” is not located at the minimal dose point.)

Step 4: Use dose profile tool in the TPS to draw a dose profile, denoted by “lineDx”, passing through “PtDx” and parallel to the tangential posterior field edge. Record the following three doses in the low‐ and high‐energy preplans in percentages of the dose at “Norm”. The subscripts “l” and “h” stand for those from the low‐ and high‐energy preplans, respectively. The same “PtDx” from low‐energy preplan is used in the high‐energy preplan.

- The maximum dose in the plan: $D_{\text{max},1},\;D_{\text{max},h}$
- The dose at point “PtDx": $D_{l},D_{h}$
- The maximum dose on the profile: $D_{\text{ml}},D_{\text{mh}}$

If the dose profile tool is not available in the TPS, other tools, such as isodose line or point dose, may be utilized to estimate $D_{\text{ml}}$ and $D_{\text{mh}}$.

Step 5: Assume that the weights for high‐ and low‐energy unblocked beams and low‐energy IMRT beams in the final hybrid plan are x, y, and z respectively, and x+y+z=1. The weights represent the sum of weights of corresponding tangential beams. For example, for the high‐energy beams, assume that a and 1−α are the weights of lateral and medial beams, respectively, determined in Step 2 (by adjusting them to balance the dose distribution along the tangential direction), then the weights of these lateral and medial high‐energy beams in the final hybrid plan are ax and (1−α)x, respectively. For IMRT beams, Eclipse TPS (Varian Medical Systems, Palo Alto, CA) always sets the weight of each beam to be one. The monitor units for each IMRT beam are determined by the inverse planning. This is reasonable because the objective constraints for inverse planning have included the goal of final dose distribution and, thus, the weight z is not necessary. For such a TPS, we need only calculate the optimal weights for the unblocked beams and then use these unblocked beams to form a sum plan as the base plan in IMRT inverse planning. As it will be clear that z is not used to calculate x and y, but instead, z is determined by x and y: z=1−x−y. For Eclipse TPS, z is introduced here just for the common sense that the sum of weights equals one.

Since we want as much dose as possible from the unblocked beams so that no primary dose is contributed from the IMRT beams (i.e., z is minimized) at the maximum dose points, we have

(1) $${\text{xD}}_{\text{max},h}+{\text{yD}}_{\text{max},1}\leq 105$$

where 105 means 105% of the prescription dose. Here we assume that dose uniformity requirement is within 95% and 105% of the prescription dose. If other numbers are used, these numbers should be replaced in the context. Equation (1) is approximate because it ignores the scatter dose from the IMRT beams at the maximum dose points, and the maximum dose points in the low‐ and high‐energy preplans may not be at the same location. The scatter dose will increase the total dose (for example, in a sum plan of the unblocked beam preplans plus the IMRT plan) at the maximum dose points, but different locations of the maximum dose points will decrease the total dose at these points.

Further, that the total dose on line “lineDx” should be within 95% and 105% of prescribed dose results in:

(2) $$x\frac{D_{h}}{D_{mh}}+y\frac{D_{l}}{D_{ml}}+z\frac{D_{l}}{D_{ml}}=x\frac{D_{h}}{D_{mh}}+(1-x)\frac{D_{l}}{D_{ml}}\geq \frac{95}{105}$$

Again, Eq. (2) ignores the match of maximum dose points on line “lineDx” in the low‐ and high‐energy unblocked preplans. It also ignores the possible different dose profile shapes in the low‐energy unblocked and IMRT beams in the final hybrid plan. However, since the same energy and beam alignment are used for the low‐energy unblocked and IMRT beams, this approximation may be acceptable.

The requirement on using as much dose as possible from low‐energy unblocked beams indicates that the inequality in 1, 2 should be replaced by equality. This is because always $D_{l}/D_{\text{ml}}<D_{h}/D_{\text{mh}}$, and thus x is minimal when

(3) $$x\frac{D_{h}}{D_{mh}}+(1-x)\frac{D_{l}}{D_{ml}}=\frac{95}{105}$$

Once the value of x is determined from Eq. (3), y becomes maximal when

(4) $${\text{xD}}_{\text{max},h}+{\text{yD}}_{\text{max},1}=105.$$

After obtaining x and y from 3, 4, the weight for IMRT beams z=1−x−y was calculated. Again, z is minimized from Eq. (1). The resulting hybrid plan is called optimal because these extreme values of weights are used. Again, for Eclipse TPS, we do not need to assign the weight of IMRT beams.

In the case where $D_{l}/D_{\text{ml}}>95$, high‐energy unblocked beams are not necessary, and low‐energy unblocked and IMRT beams are sufficient to obtain the expected dose distribution. This happens in small breast and small chest wall cases, namely, with small lateral separations along beam direction. On the other hand, if $D_{h}/D_{\text{mh}}<95$, even higher energy photon beams are needed.

In the case where x=0, there is no mismatch of the maximum dose points in the low‐ and high‐energy unblocked beams. The scatter dose from the IMRT beams will increase the dose at the maximum dose point in the final hybrid plan. The value of y from Eq. (4) should be decreased a little (for example, 3%), in order to keep the maximum dose within 105%.

The values of x and y mainly depend on the maximum separation of the breast or chest wall, but the shape of target also affects the dose distribution and, thus, the values of x and y.

In a case where “PtDx” is not at the minimal dose point, the sensitivity of x on the relative dose at “PtDx” can be analyzed from Eq. (3). Generally, the sensitivity is inversely proportional to $\frac{D_{h}}{D_{\text{mh}}}-\frac{D_{l}}{D_{\text{ml}}}$ Small breasts/chest walls usually have smaller values of $\frac{D_{h}}{D_{\text{mh}}}-\frac{D_{l}}{D_{\text{ml}}}$, thus the value of x is more sensitive to the relative dose at “PtDx” in small breast/chest wall cases. For example, for a small breast case with maximal separation of 21 cm (“breast B” in Table 1), where “PtDx” was 2 cm and 2.7 cm from the posterior edges of tangential beams, the relative difference of the point doses was 1.5%, and the relative difference of the value of x was 31%. For a big breast case with maximal separation 28 cm (“breast E” in Table 1), where “PtDx” was 2 cm and 2.3 cm from the posterior edges of tangential beams, the relative difference of the point doses was 1.5%, and the relative difference of the value of x was 19%. Further, “PtDx” will affect the final dose distribution. If “PtDx” is not at the minimal dose point, but n% higher than the minimal dose in the preplans, then the final minimal dose will be roughly n% lower than 95% of prescribed dose in the hybrid plan.

**Table 1:** Optimal weights, x, y, and z to 18 and 6 MV unblocked beams and 6 MV IMRT beams, respectively, for 11 breast/chest wall cases with various maximum separations. Skin was considered as part of the target for all the cases (the optimal procedure was conducted by this assumption, even though for “breast F” the physician contoured CTV was 5 mm away from the body contour). The jaw opens refer to the tangential beams only, and the prescribed dose to supraclavicular nodes is not listed

*Patient*	*Prescribed Dose (cGy)*	*Max. Separation (cm)*	*Jaw Opens (x, y) in cm*	*Bolus (mm)*	*x*	*y*	*z*
breast A	266 X 16	21	(12.6, 19.0)	0	0.00	0.91	0.09
breast B^a^	266 X 16	21	(12.2, 20.7)	0	0.35	0.62	0.03
breast C	266 X 16	23	(13.6, 19.3)	0	0.46	0.5	0.04
breast $D^{\text{ab}}$	200 X 25	24	(21.9, 19.0)	0	0.33	0.64	0.03
breast E	267 X 15	28	(12.4, 20.0)	0	0.70	0.24	0.06
breast F	267 X 15	15	(8.3, 20.5)	0	0.00	0.99	0.01
chest wall A^b^	200 X 25	18	(13.9, 20.0)	4	0.00	0.89	0.11
chest wall B^b^	200 X 25	20	(12.1, 15.7)	4	0.26	0.64	0.10
chest wall C^b^	200 X 25	23	(15.2, 15.0)	4	0.31	0.60	0.09
chest wall D^b^	200 X 25	23	(10.0, 21.0)	4	0.50	0.40	0.10
chest wall E^b^	200 X 25	25	(12.0, 17.0)	4	0.56	0.37	0.07

a Auxillary nodes involved.

b Supraclavicular nodes involved.

Once we obtain the values of x and y from the unblocked beam preplans, an unblocked beam sum plan composed of the high‐ and low‐energy unblocked beam preplans is generated with weights x and y, respectively. If the target volume covers the whole breast/chest wall region determined by the beam apertures set by the physician, we can construct the PTV for IMRT planning from the unblocked beam sum plan — for example, as the 55% isodose volume cropped from lung, heart, and body (for intact breast) or bolus (if bolus is added) with a few millimeters margin. At our institute, the aperture setup followed the recommendation of RTOG 1005. We found that use of the 55% isodose volume as the base of PTV resulted in satisfied dose coverage. Thus, unless PTV was delineated by physicians, the PTV was constructed in this way throughout this work. Finally, we copy the low‐energy unblocked beams as our initial IMRT beams (including the same gantry angle and collimator angle) and use the unblocked beam sum plan as the base plan for the inverse planning of the IMRT beams. If supraclavicular nodes are involved, the base plan consists of the unblocked beam preplans and the supraclavicular plan. In order to spare the lung and contralateral breast, the jaws in the IMRT fields are fixed to prevent adjustment by the inverse planning algorithm.

For the inverse planning, we set two objectives for the PTV, Lower: 100% volume to get 95% prescription dose with priority 100, and Upper: 0% volume to get 105% prescription dose with priority 200. The higher priority for Upper than Lower is to avoid monitor units dumping onto skin. Objectives for other involved structures were set with priority of 50. For example, 100% of the volume of seroma gets at least full prescription dose, and 30% of the volume of ipsilateral lung gets at most 20 Gy. Jaws were fixed during optimization. The planning usually reaches the optimal result within 50 iterations. The inversely planned IMRT fluence map usually has zero fluence in the skin region. In this case, skin flash for the IMRT beams is not necessary.

The above discussion is for situations in which the skin is part of the target. For other cases, however, the skin is not the target or is oversensitive to radiation, so that we may want to minimize skin dose.^(2)^ For such cases, from Eq. (1) we have the maximal $x=105/D_{\text{maxh}},\;\text{and}\;y=0,\;z=1-x$. In a case where $D_{\text{maxh}}<105$ (i.e., 105% of the prescription dose), there are two situations: (1) if $D_{h}\geq 95$, the high‐energy beams alone can cover the target with expected dose and no IMRT beams are needed; otherwise, (2) if $D_{h}<95$, the dose normalization point “norm” should be reselected so that $D_{h}>95$. After renormalization, if $D_{\text{maxh}}\leq 105$, no IMRT beams are needed, and otherwise, the optimal weights are $x=105/D_{\text{maxh}},\;y=0,\;\text{and}\;z=1-x$.

In this work, we mainly focus on the case that the skin is included in the target.

### B. Quantitative evaluation of plans

The following indices were used in evaluating PTV dose coverage. The dose in the ipsilateral lung (i.e., the main organ at risk for breast and chest wall radiation treatment) was indexed by $V_{20}$, the percentage volume of ipsilateral lung receiving at least 20 Gy dose in the course.

- Dose uniformity index (UI):^(14)^ $\text{UI}=D_{5}/D_{95}$
- Dose homogeneity index (HI):^(15)^ $(D_{1}-D_{99})/\text{Rx}.$
- Dose conformity index (CI):^(16)^ $\text{CI}=V_{\text{RI}}/\text{TV}$

Here, Dj represents the minimal dose received by 1% of PTV, and so for $D_{5},D_{95}$, and $D_{99}$. Rx is the prescribed dose. $V_{\text{RI}}$ is the volume of PTV covered by the reference isodose line, which we used 95% isodose line in this work. TV is the target volume, which was PTV in our case, except one breast case for which the physician contoured CTV and thus we used the CTV as the target volume.

### C. Treatment planning system and patient data

Eclipse 10.0 TPS and the AAA 10.0 dose calculation algorithm were used. The system and the millennium 120 MLC were commissioned for sliding window technique. The low‐ and highphoton energies were 6 and 18 MV, respectively.

Six intact breast and five chest wall patient cases were studied, including one breast case for which the physician contoured CTV so that our optimal algorithm could be used to compare patient to patient. 4 mm bolus was added onto the chest wall, but no bolus for the breast patients. The maximum separation of the breast and chest wall including bolus was from 15 cm to 28 cm.

### D. Verification of breathing effect

To check the breathing effect, for simplicity and without loss of generality, we assumed the patient was set up at the middle breathing status. The verification plan was generated in Eclipse with a slab of 5 cm solid water on a MapCHECK device (MapCHECK2, Sun Nuclear Corp., Melbourne, FL) and the angles of the gantry, collimator and couch all were set to zero degrees. This is our standard setup for individual IMRT QA for head and neck and prostate plans. The planned and delivered doses from both unblocked and IMRT beams on the detector plane were compared. During dose delivery, the MapCHECK was fixed on an XY/4D motion‐simulation table (Sun Nuclear Corp.). The table was moving laterally and periodically to simulate patient breathing with amplitude ±0.25,±0.5,±1cm for shallow, normal, and deep breathing patterns, respectively. The breathing pattern was reconstructed from the probability density function of a lung patient's breathing wave, which was recorded by our RPM facility (Varian Medical System).

Breast movement with breathing is not in 1D but typically in 3D. Because the fluence change is the most pronounced along the direction perpendicular to the skin, our QA setup detects the most significant breathing effect. Depending on individual breathing movement, actual breathing effect may not be as significant as that measured in this experiment.

## III. RESULTS

### A. Optimal weights

Table 1 lists the values of x, y, and z determined from our optimization approach (i.e., 3, 4) for six breast patients and five chest wall patients with different maximum separations and treated with 6 and 18 MV tangential unblocked beams and 6 MV tangential IMRT beams. For the cases x=0, the listed value of y was directly obtained from these equations without adjustment. As discussed above, when x=0, the value of y may need to decrease a little so that the maximal dose will be kept at about 105% of prescribed dose. In our planning, when x=0, the value of y used in plans was 3% lower than that listed. It is seen that z is not greater than 0.1 except for a small chest wall. This indicates that for most cases, more than 90% dose will be delivered from unblocked beams, and thus efficiently limits the breathing effect. Further, the values of x, y, z are not uniquely determined by the maximum separation, but roughly x increases with the maximum separation.

### B. Results from treatment plans

**Figure 2:**
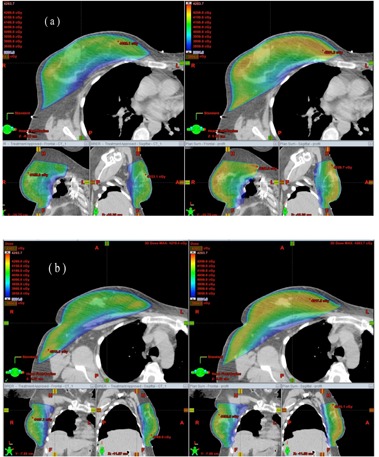
Dose distribution on the transversal, frontal, and sagittal planes passing point (a) “Norm” and (b) “PtDx” (as indicated by the crossed lines) for patient “breast E”. The left side is from the 3D CRT plan and the right side is from the optimal hybrid plan.

**Figure 3:**
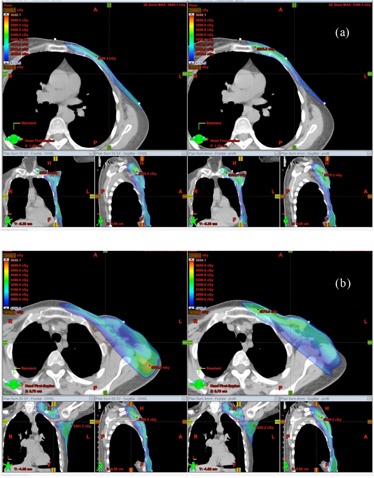
The same as Fig. 2 but for patient “chest wall D”. The structure node is displayed for the dose coverage.

**Figure 4:**
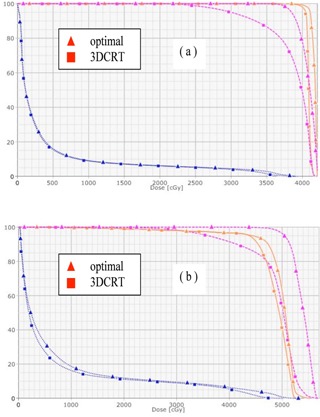
DVHs of PTV (in dashed line), ipsilateral lung (in points), and seroma/node (in solid line) in the 3D CRT (in square) and optimal hybrid (in triangle) plans for patients (a) “breast E” and (b) “chest wall D”.

Figures 2(a) and 2(b) display the dose distributions on the transversal, frontal, and sagittal planes passing the points “Norm” and “PtDx”, respectively, for patient “breast E”. Figure 3 displays a chest wall plus supraclavicular case (patient “chest wall D”). 4 mm bolus was added onto the chest wall for the tangent beams, and Rx=200cGyx25 for both the chest wall and supraclavicular nodes. Figure 4 displays the DVHs of PTV, ipsilateral lung, and seroma/node. It is seen that, compared with 3D CRT plans, the optimal hybrid plans gave much better dose coverage on PTV including skin, seroma, and involved nodes. (Note: The seroma is not displayed in Fig. 2 because it is not in the planes passing the points “Norm” and “PtDx”.) For the hybrid plans, the dose in the breast and chest wall was between 95% and 107% of the prescription dose. In the chest wall plan, the IMRT beams caused some hot spots (< 108%) in the supraclavicular side, while in the 3D CRT plan, hot spots (< 112%) appeared on both chest wall and supraclavicular sides. The maximum dose in the hybrid plan was greater than 105% because of the scatter dose from the IMRT beams.

Table 2 lists the quantitative comparison of dose uniformity index (UI), homogeneity index (HI), and conformity index (CI) from our optimal hybrid and 3D CRT plans for these 11 patient cases. The ipsilateral lung dose index, $V_{20}$, is also listed in the table, as lung is the main organ at risk for breast and chest wall treatment. From UI, HI, and CI viewpoint, the hybrid plans gave systematically better result than 3D CRT plans, except the plan for patient “chest wall A”, where these indices had the similar values. For this patient, x=0, so that it is relatively easy to make a 3D CRT plan comparable to the optimal hybrid one. The lung dose from the hybrid plans was comparable to that from the 3D CRT plans for all these breast and chest wall cases.

One challenge of 3D CRT planning is to remove cold spots at the beam junction. When different energy beams are used for breast (chest wall) and supraclavicular nodes, the halfbeam profiles cannot merge perfectly at the junction. As shown in Fig. 3, the hybrid planning successfully overcame the beam junction issue.

**Figure 5:**
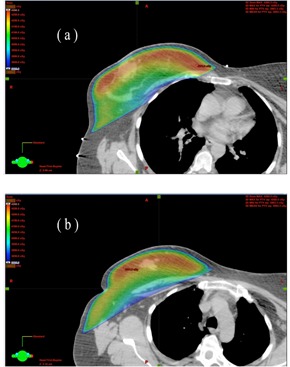
DVHs of PTV (in dashed line), skin (in solid blue line), ipsilateral lung (in points), and seroma (in solid brown line) in the optimal (in square) and nonoptimal (in triangle) hybrid plans for patient “breast E”.

**Figure 6:**
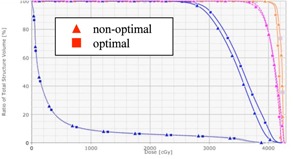
DVHs of PTV (in dashed line), skin (in solid blue line), ipsilateral lung (in points), and seroma (in solid brown line) in the optimal (in square) and nonoptimal (in triangle) hybrid plans for patient “breast E”.

**Figure 7:**
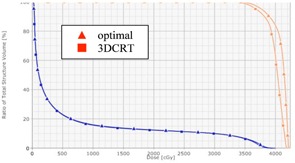
DVHs of CTV (in brown) and ipsilateral lung (in blue) in the optimal (in triangle) and 3D CRT (in square) plans for patient “breast F”. The CTV was contoured by the physician and used as the target volume in inverse planning. No PTV was contoured in both plans.

If optimal weights are not used in the planning, the resulting dose distribution is not optimal ( e.g., cold spots in the target, more skin sparing, and high IMRT fluence near skin). Figure 5 displays the dose distribution from a hybrid plan for patient “breast E” with x=0.7,y=0,andz=0.3. All the other parameters were the same as those used for the optimal plan, including the beam geometry, PTV, and objective constraints for inverse planning, and so on. Figure 6 shows the DVHs of PTV, skin, lung, and seroma in the optimal and nonoptimal hybrid plans for patient “breast E”. The skin structure was contoured to be 5 mm thick from the body around the PTV region. Compared with those in the optimal plan, the skin dose in the nonoptimal plan was more spared and the IMRT fluence (see Fig. 7) nearby the skin was high. The AAA algorithm may not give highly accurate dose calculation on the skin region,^(17)^ but physically it is true that low‐energy unblocked beams deliver more dose onto skin than high‐energy unblocked beams do, and inverse planning can result in more skin sparing.

While the target (PTV) in all the other cases was constructed from the sum plan of the unblocked preplans, the target (CTV) in “breast F” was contoured by the physician and used in the original 3D CRT planning. This allowed us to have a more restrictive comparison of the 3D CRT plan with our hybrid plan. The CTV covered the whole breast but was about 5 mm away from the body contour. The CTV was used as the target volume in the inverse planning (i.e., no PTV was constructed from the sum plan of the unblocked preplans). All other optimal procedures were the same as those for other breast and chest wall cases, and the same objective constraints for PTV in other cases were used for the CTV in this case. Figure 7 displays the DVHs of the CTV and ipsilateral lung from the original 3D CRT plan and our optimal plan. It is seen that the CTV dose coverage was much better from the optimal plan than from the 3D CRT one, and the lung doses from these two plans were similar.

**Table 2:** Dose uniformity index (UI), homogeneity index (HI), conformity index (CI), and ipsilateral lung dose index $V_{20}$ from the optimal hybrid and 3D CRT plans for the patients listed in Table 1. The target for “breast F” was the CTV contoured by the physician, and the target for the others was the PTV constructed from the preplans. The values from the 3D CRT plans are given in parentheses

*Patient*	*UI*	*HI*	*CI*	$\text{Lung}\;V_{20}(\%)$
breast A	1.06(1.09)	0.10(0.12)	0.99(0.96)	8.3(8.0)
breast B^a^	1.09(1.10)	0.12(0.16)	0.97(0.96)	17.7(17.8)
breast C	1.09(1.14)	0.11(0.19)	0.95(0.84)	9.0(8.1)
breast $D^{\text{ab}}$	1.09(1.11)	0.12(0.17)	0.95(0.91)	20.3(19.5)
breast E	1.05(1.38)	0.12(0.43)	0.97(0.73)	6.2(6.1)
breast F	1.12(1.13)	0.16(0.18)	0.94(0.91)	12.4(12.3)
chest wall A^b^	1.06(1.07)	0.09(0.09)	0.99(0.99)	27.0(27.2)
chest wall B^b^	1.12(1.54)	0.18(0.57)	0.94(0.81)	26.0(26.0)
chest wall C^b^	1.09(1.52)	0.12(0.55)	0.97(0.76)	21.0(21.2)
chest wall D^b^	1.11(1.49)	0.15(0.54)	0.94(0.79)	11.5(11.2)
chest wall E^b^	1.11(1.70)	0.20(0.59)	0.94(0.70)	25.7(22.5)

a Auxillary nodes involved.

b Supraclavicular nodes involved.

### C. Results from verification plans

**Figure 8:**
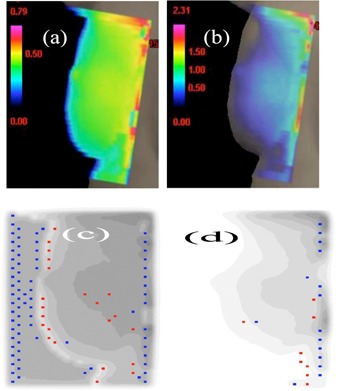
IMRT fluence map ((a) and (b)) from the medial IMRT beam in a nonoptimal hybrid plan (with the same parameters as in Fig. 5) and in the optimal hybrid plan, respectively. Breathing‐caused dose discrepancy map (with amplitude 1 cm) ((c) and (d)) between planned and delivered doses from the medial IMRT beam in the verification plan of a nonoptimal plan (with the same parameters as in Fig. 5) and of the optimal hybrid plan, respectively, for patient “breast E”. The red spots indicate higher dose delivered than planned from 3%/3 mm limits (gamma>1), and the blue spots for lower dose delivered and from the same limits. Skin flash was applied to the nonoptimal plan for Fig. 8(c) but not displayed in Fig. 8(a) in order to show the fluence difference between optimal and nonoptimal IMRT beams.

We checked the breathing effect on the optimal and nonoptimal hybrid plans. As a typical example, here we give the results from patient “breast E”. Figures 8(a) and (b) display the IMRT fluence maps of the optimal and nonoptimal IMRT plans. The body contour indicates the skin place. It is seen that the fluence of the optimal plan somewhat smoothly decreases from the posterior field edge and is close to zero near the skin. For the optimal plan, no skin flash was added for examining the breathing effect. For the nonoptimal plan, the fluence from the inverse planning was high near the skin region and thus skin flash was necessary to decrease the breathing effect. The fluences displayed in Figs. 8(a) and (b) were purely from the inverse planning in order to compare the fluence difference between optimal and nonoptimal IMRT beams, but before generating the verification plan, skin flash was applied to the nonoptimal IMRT fluence. Figures 8(c) and (d) display the breathing effect on the medial IMRT beams with a breathing wave oscillating between −1and1cm. The XY/4D motion‐simulation table was positioned at 0 cm before introducing the oscillation. No skin flash was used to the optimal beams (Fig. 8(d)) but applied to the nonoptimal ones (Fig. 8(c)). Figure 8 indicates that, compared with the nonoptimal IMRT beam, the breathing effect on the optimal IMRT beam was much alleviated, especially on the skin region.

Table 3 lists the gamma test pass rates for the whole tangential beams (unblocked plus IMRT beams) in the optimal and nonoptimal plans when the breathing wave oscillated between ±0.25,±0.5,and±1cm, respectively. The pass rates were similar when the breathing amplitude was small, but the optimal plan outperformed when the breathing amplitude was large.

**Table 3:** Gamma test (3%/3 mm and 10% dose threshold) pass rate of the verification plans (including the unblocked and IMRT beams) for patient “breast E” under various breathing amplitudes

*Whole Tangential Beams*	*Nonoptimal Lateral*	*Nonoptimal Medial*	*Optimal Lateral*	*Optimal Medial*
static	99.8	99.9	100	100
5 mm breath	99.4	99.6	99.9	100
10 mm breath	98.2	98.8	99.5	99.7
20 mm breath	90.7	91.1	94.7	94.9

## IV. DISCUSSION

Besides breathing effect, there are other concerns on adopting IMRT for breast treatment including interfraction patient setup uncertainty, skin dose, and total monitor units used in the course. Without breathing factor, it has been found that the setup uncertainty results in worse dose delivery by IMRT than 3D CRT because the modulated fluence in IMRT is more sensitive to patient positioning than the relatively uniform fluence in 3D CRT.^12^, ^13^ Because of the wobbling nature of the target, patient positioning may thus pose a challenge when using IMRT.^(18)^ Our approach optimally assigns minimal weight to the IMRT beams, and thus may help alleviate the effect of positioning uncertainty. Particularly, when IMRT fluence is low in the wobbling region, the wobbling effect is minimized.

Compared to breathing control techniques such as breath holding and gating, preventive design from treatment planning would be an economic and efficient way. The result of breathing control techniques largely depends on the patient status during dose delivery. Breath‐holding technique may not be suitable for each patient, and gating practice requires 4D CT techniques and suffers longer treatment time. In contrast, our optimal planning is generally patient status independent and no extra facility and techniques are required. The time involved in the planning is also shortened compared with the time for 3D CRT planning. It takes about 1 to 2 hours per hybrid plan from the setup of the preplans to the complete of the final hybrid plan, while often more than 2 hours for a 3D CRT plan, depending on the planner's experience.

In this work, the IMRT beams use the same beam geometric parameters as the unblocked beams, such as the gantry angles, collimator angles, and jaw openings. This is because we want to show the advantage of our optimal planning over 3D CRT and nonoptimal ones. However, it may not be necessary to have the same geometric parameters. Also in this work, we consider only two extreme cases: the skin dose is required either as high as or as low as possible. Physicians may ask for neither of these but for something in between. In this case, the optimal procedure may become more complicated and needs further investigation. Furthermore, our approach can be also used in the optimal beam weight calculation for 3D CRT plans consisting of unblocked beams and field‐in‐field segments. In this case, the weight for IMRT beams becomes the weight for the field‐in‐field segments. Such an optimal 3D CRT planning can improve dose uniformity and save planning time. The result will be published elsewhere.

## V. CONCLUSIONS

We have proposed a two‐point approach to calculating optimal weights of beams in hybrid breast plans consisting of tangential low‐ and high‐energy unblocked beams and low‐energy IMRT beams. These two points are the normalization point and the minimal dose point in the preplan of low‐energy unblocked beams. From these two points and a line passing through the minimal dose point and parallel to the posterior field edges of the tangential beams, the maximum and minimum doses in the preplans are recorded and used to calculate the optimal beam weights in the final hybrid plan. The goal of our optimization is to effectively decrease the breathing effect and to keep the skin dose as much as possible if the skin is included in the target. This requires that maximal weights be assigned to the unblocked beams, especially the low‐dose unblocked beams, while the IMRT fluence being zero near the skin. From the treatment plans and verification measurements, we have shown that our optimal hybrid plans have advantages over nonoptimal ones in mitigating breathing effect and keeping the skin dose, and meanwhile hold the advantages of IMRT plans over 3D CRT plans in dose uniformity and conformity, particularly in beam junction. This approach can be adopted to satisfy other treatment requirements too — for example, by adjusting the weights of the low‐energy unblocked and IMRT plans to spare skin dose.

## ACKNOWLEDGMENTS

The author acknowledges the fruitful discussions with Ms. Maria Corsten, Mr. Tim Healey at Eastern Health, Dr. Shuying Wan at Sudbury Hospital, and Dr. Jun Li at Thomas Jefferson University. Also thanks to the associate editor and anonymous reviewers for their dedicated comments.
